# lncRNASNP: a database of SNPs in lncRNAs and their potential functions in human and mouse

**DOI:** 10.1093/nar/gku1000

**Published:** 2014-10-20

**Authors:** Jing Gong, Wei Liu, Jiayou Zhang, Xiaoping Miao, An-Yuan Guo

**Affiliations:** 1 Department of Epidemiology and Biostatistics, School of Public Health, Tongji Medical College, Huazhong University of Science and Technology, Wuhan, Hubei 430030, PR China; 2 Department of Biomedical Engineering, Key Laboratory of Molecular Biophysics of the Ministry of Education, College of Life Science and Technology, Huazhong University of Science and Technology, Wuhan, Hubei 430074, PR China; 3 Department of influenza vaccine research, Wuhan Institute of Biological Products, Wuhan, Hubei 430207, PR China

## Abstract

Long non-coding RNAs (lncRNAs) play key roles in various cellular contexts and diseases by diverse mechanisms. With the rapid growth of identified lncRNAs and disease-associated single nucleotide polymorphisms (SNPs), there is a great demand to study SNPs in lncRNAs. Aiming to provide a useful resource about lncRNA SNPs, we systematically identified SNPs in lncRNAs and analyzed their potential impacts on lncRNA structure and function. In total, we identified 495 729 and 777 095 SNPs in more than 30 000 lncRNA transcripts in human and mouse, respectively. A large number of SNPs were predicted with the potential to impact on the miRNA–lncRNA interaction. The experimental evidence and conservation of miRNA–lncRNA interaction, as well as miRNA expressions from TCGA were also integrated to prioritize the miRNA–lncRNA interactions and SNPs on the binding sites. Furthermore, by mapping SNPs to GWAS results, we found that 142 human lncRNA SNPs are GWAS tagSNPs and 197 827 lncRNA SNPs are in the GWAS linkage disequilibrium regions. All these data for human and mouse lncRNAs were imported into lncRNASNP database (http://bioinfo.life.hust.edu.cn/lncRNASNP/), which includes two sub-databases lncRNASNP-human and lncRNASNP-mouse. The lncRNASNP database has a user-friendly interface for searching and browsing through the SNP, lncRNA and miRNA sections.

## INTRODUCTION

The ENCODE project shows that most of the human genome nucleotides can be transcribed into primary transcripts in different cells (1), producing a range of non-coding RNAs (ncRNAs). Long non-coding RNAs (lncRNAs) are ncRNAs defined as transcripts >200 nt in length, which are typically transcribed by RNA polymerase II and are often multiexonic and polyadenylated (2). lncRNAs play critical roles in various biological processes and cellular contexts by diverse mechanisms to participate in the regulation of transcription/post-transcription/post-translation, organization of protein complexes, cell–cell signaling as well as recombination (3,4). The functions of lncRNAs are described in terms of their expressions, biological processes, diseases mechanisms, as well as their polymorphisms (5). By dysregulating target gene expression, lncRNAs involve in a number of complex human diseases, including coronary artery diseases, autoimmune diseases, neurological disorders and various cancers (6).

Single nucleotide polymorphisms (SNPs) represent the most frequent genetic variants among individuals and link to gene expression, function, phenotypes and diseases (7). Recently, SNPs in lncRNAs were found to be linked to their abnormal expressions and dysregulations, thus play important roles in disease association (5,8). For example, the rs2839698 TC genotype of lncRNA H19 was associated with a low risk of developing non-muscle-invasive bladder cancer (9). SNP rs6983267 affects the expression of lncRNA CCAT2 thus impacts tumor growth and metastasis in colorectal cancer (10). In addition, many genome-wide association studies (GWASs) have identified several trait-associated SNPs in or nearby lncRNAs, such as antisense noncoding RNA in the INK4 locus (ANRIL) (11). Therefore, it is important to identify SNPs in lncRNAs, especially link SNPs in lncRNAs with GWAS results for studying complex traits and diseases.

MicroRNAs (miRNAs) are another class of small ncRNAs. lncRNA can interact with miRNA by complementary sequence to act as a miRNA decoy or sponge, which are widely confirmed by experiments (12–15). For example, lncRNA PTENP1 function as a decoy for PTEN-targeting miRNAs in tumor suppression (14). Currently, starBase has collected more than 10 000 miRNA–ncRNA interaction pairs from high-throughput studies (16). SNPs in miRNA target sites on lncRNAs may influence (destroy or create) the miRNA–lncRNA interactions, thereby alter their functions. For protein-coding genes, SNP impacts on their miRNA target sites has been validated in many studies (17,18) and a number of databases for predicting functional SNPs in miRNA target sites were constructed, including MicroSNiPer, PolymiRTS, miRdSNP, MirSNP and our previous work miRNASNP (19–23). Thus, a database for SNPs in lncRNAs and their potential functions on lncRNA–miRNA binding will be very useful to accelerate the researches.

Currently, there are many databases about lncRNA sequence, expression and function, including NONCODE, LNCipedia, lncRNAdb, lncRNAtor, lncRNAMap, starBase, lncRNome, Linc2GO and LncRNADisease (16,24–31). There are very few studies and databases about the lncRNA SNPs on genome-wide, which include systematical analyses of SNPs in long intergenic noncoding RNAs (lincRNAs) and a database named LincSNP linking disease-associated SNPs to human lincRNAs (32,33). However, both studies focused on lincRNAs, a subclass of lncRNAs and both of them didn't analyze the SNP effects on miRNA–lncRNA interactions. Until now, there is no genome-wide analysis or a database for the comprehensive lncRNA related SNPs and their potential functions.

In this study, we constructed the lncRNASNP database aiming to provide comprehensive information about lncRNA related SNPs in human and mouse and explore their potential functions. We characterized all SNPs in lncRNA exon sequences, and predicted their effects on lncRNA secondary structure and lncRNA–miRNA interaction. Furthermore, we identified human lncRNA SNPs in the GWAS linkage disequilibrium (LD) regions to link lncRNAs to phenotypes. The lncRNASNP has a user-friendly interface for searching and displaying. We hope it will be a useful resource for the lncRNA, SNP and miRNA research communities.

## DATA COLLECTION AND DATABASE CONTENT

### SNPs in lncRNA transcripts

The human and mouse SNP data were downloaded from dbSNP of NCBI (human GRCh37 and mouse mm10). We obtained the human lncRNA data from LNCipedia database, which contains 32 108 human lncRNA transcripts of 17 436 lncRNA genes (GRCh37) (25). We extracted 36 471 confident mouse lncRNA transcripts from NONCODEv4 by excluding the unpublished and unconfident data. The genomic coordinates of mouse lncRNAs were converted from genome assembly mm9 to mm10 using the LiftOver utility in UCSC (http://genome.ucsc.edu). By comparing the genomic coordinates of SNPs with those of lncRNAs, we identified 495 729 SNPs and 777 095 SNPs in human and mouse lncRNA transcripts (Table 1), respectively. We refer to these SNPs as lncRNA SNPs in human and mouse.

**Table 1.** Data summary in lncRNASNP database

Data content	Number of records	
	Human	Mouse
lncRNA gene/transcript	17436/32108	25512/36471
miRNA	2576	1900
SNPs in lncRNA transcripts	495729	777095
SNPs affect MLP^a^ (loss/gain)	262154/280012	366731/357246
All predicted MLP	6413273	7448200
Conserved MLP^b^	69837	13780
Experimental MLP^c^ (all/loss by SNPs)	8091/2116	NA
MLP affected by SNPs (loss/gain)	799694/830829	898220/864767
MLP loss by SNPs with miRNA RPM>1^d^	158105	NA
MLP gain by SNPs with miRNA RPM>1	165731	NA
lncRNA SNPs are GWAS tagSNPs	142	NA
lncRNA SNPs in GWAS LD regions	197827	NA

^a^MLP means miRNA-lncRNA target pair.

^b^The miRNA binding sites are existed in all the conserved exons across human, mouse, rat, and dog.

^c^The miRNA–lncRNA target pairs supported by CLIP experiment results from starBase.

^d^The miRNA RPM is the average value estimated from TCGA samples (9566 samples across 28 cancer types).

### Impacts of SNPs on lncRNA structures

A part of lncRNAs can exert their functions by scaffolding protein complexes (34), suggesting the structural features of lncRNAs play important roles in their functions. SNPs in lncRNA transcripts may influence the lncRNA secondary structure to impact its stability, expression and functions (35). To assess the impacts of SNPs on lncRNA secondary structures, we first extracted lncRNA transcript sequences from human/mouse reference genome according to the lncRNA transcript BED file as Ref-transcripts. For each SNP, we changed the corresponding allele in the given Ref-transcript to alternative allele as Alt-transcript. Then, RNAfold program (36) was used to illustrate the secondary structure and calculate the minimal free energy (MFE, Δ*G*). Energy change of RNA structures (ΔΔ*G*) was calculated by the MFE differences using ΔΔ*G* = |ΔGalt − ΔGref|, where ΔGref and ΔGalt are the MFEs of the Ref-transcript and Alt-transcript, respectively. In human and mouse lncRNAs, the average energy changes by SNPs are (1.30 ± 1.62) kcal/mol and (1.26 ± 1.27) kcal/mol, while the energy changes of the top 10% are 3.10 kcal/mol and 3.00 kcal/mol, respectively.

### miRNA–lncRNA interaction

Increasing evidence shows that lncRNAs can be directly regulated by miRNAs (16), thereby indirectly regulate gene expression by competing with shared miRNAs (12). In lncRNASNP database, we not only predicted miRNA target sites on lncRNAs, but also integrated the interaction conservation, miRNA expression and experimental validated miRNA–lncRNA interaction for prioritization.

After downloading mature miRNA sequences from latest version of miRBase (37) (release 20), miRNA target sites on lncRNAs were predicted by combining the results of two popular tools, namely TargetScan (38) and miRanda (39). To further reduce false positives and prioritize the interactions, we also considered the conservations of target sites and the expression of miRNAs. The miRNA binding sites existing in the conserved exons across human, mouse, rat and dog were classified as conserved. The conservation analysis of lncRNA exons were analyzed by UCSC LiftOver tool and set the parameter of ‘minimum ratio of bases that must remap’ as 0.5. By analyzing the miRNA expression profiles of 9566 human small RNA samples across 28 diseases from The Cancer Genome Atlas (TCGA), we found that only 553 miRNAs (21%) have an expression level >1 RPM (reads per million) and the remaining 79% miRNAs are almost no expression. In a specific tissue, the predicted bindings of these unexpressed miRNAs may be very week and useless. Thus, we provided the miRNA expression levels to help users to assess the confidence of the miRNA–lncRNA interaction in specific tissues. Furthermore, we also included 8091 experimentally supported human miRNA–lncRNA interactions from starBase (16).

### Impacts of SNPs on miRNA–lncRNA interactions

SNPs in the miRNA target sites may destroy or create miRNA binding sites on lncRNAs, which result in loss and/or gain function of miRNA–lncRNA interactions. To assess the impact of each SNP on miRNA–lncRNA interaction, we used TargetScan and miRanda to predict target sites on the SNP around sequences (±25 bp around SNP) of Ref-transcripts and Alt-transcripts. Thus, we obtained four groups of target datasets, which were recorded as RT (targets on Ref-transcripts by TargetScan), RM (targets on Ref-transcripts by miRanda), AT (targets on Alt-transcripts by TargetScan) and AM (targets on Alt-transcripts by miRanda). The miRNA–lncRNA pairs exist in both RT and RM, but in neither AT nor AM, were defined as interaction losses. On the contrary, if a miRNA–lncRNA pair was predicted in both AT and AM, but in neither RT nor RM, we defined the Alt-transcript gained a novel target site. We found a large number of SNPs with the potential to disturb original miRNA target sites or/and created new potential miRNA target sites. The detail results are shown in Table 1.

### SNPs in GWAS identified trait associated regions

Currently, GWASs have linked more than 10 000 SNPs to human traits and diseases. However, most of them locate outside of protein-coding regions. The lncRNASNP database attempts to link SNPs in lncRNAs to phenotypes and human diseases. We utilized the GWAS results from the NHGRI GWAS Catalog (40). By intersecting the GWAS identified strongest risk SNPs (record as GWAS tagSNPs in our study) with SNPs in lncRNAs, we found 142 GWAS tagSNPs in human lncRNAs. Aim to better utilize the GWAS data, we also captured the LD blocks of each tagSNP for different populations using Haploview (setting *R*^2^ ≥ 0.8) (41) by analyzing the genotype information of ±500 kb around the tagSNP and defined these LD blocks as GWAS identified trait associated regions. The used populations include ASW, CHB, CEU, CHD, JPT, TSI, YRI, LWK, MEX, MKK and GIH. Our analysis shows that 197 827 lncRNA SNPs are in the GWAS LD regions of all populations.

## DATABASE ORGANIZATION AND WEB INTERFACE

**Figure 1.**
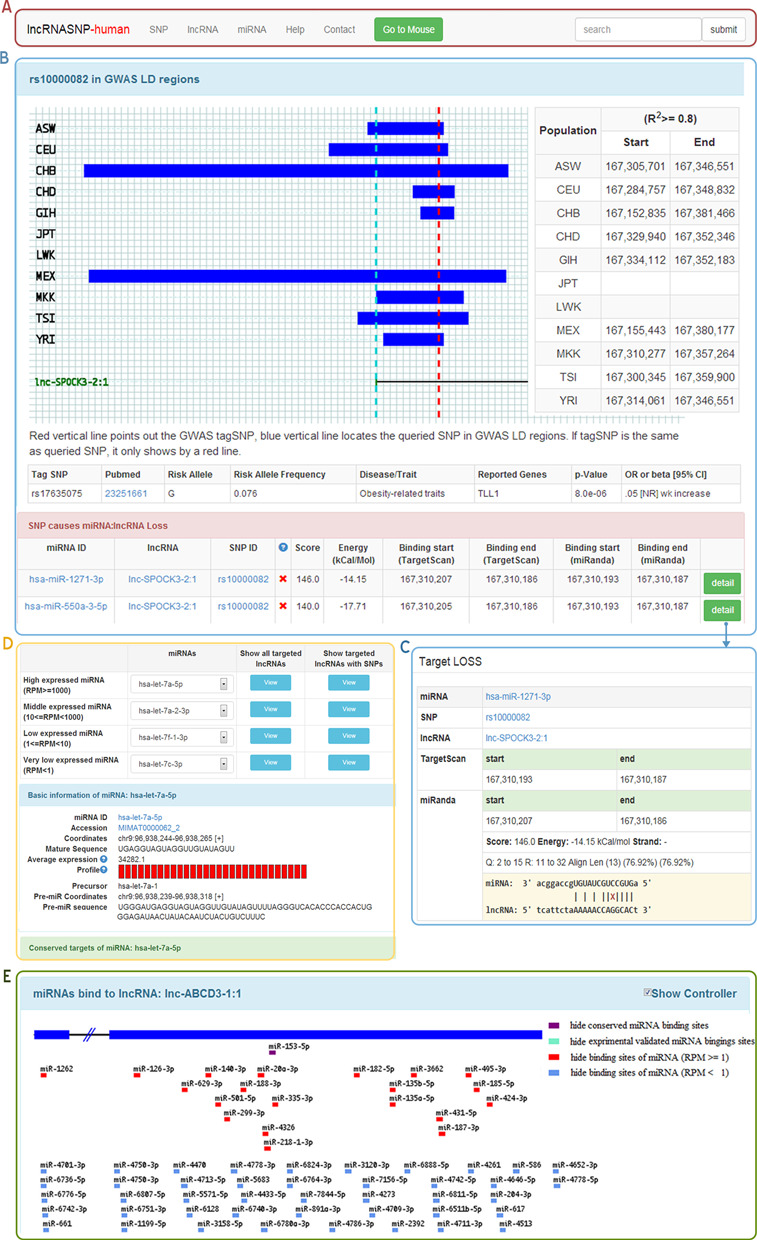
Overview of the lncRNASNP database. (**A**) The navigation bar of the database, including three main sections. (**B**) The SNP page with GWAS information and SNP effects on miRNA–lncRNA interactions. (**C**) An example of miRNA target loss by a SNP in lncRNA. (**D**) The miRNA section to search all targeted lncRNAs of miRNAs in four categories based on their expression. (**E**) The miRNA binding sites on a lncRNA.

All data were organized into a set of relational MySQL tables. The lncRNAs transcript ID, miRNA ID and SNP ID were used as the primary keys to organize and link all tables. The lncRNASNP website (http://bioinfo.life.hust.edu.cn/lncRNASNP/) was built with the Ruby on Rails (RoR) open-source web framework. We developed several user-friendly search boxes to make the data retrieval easily and efficiently. A quick search box was designed at the top-right of each page for searching by lncRNA transcript ID, miRNA ID, SNP ID and even a defined chromosome region (Figure 1A). The advanced search was provided on the home page with multiple ways for searching by different keywords or batch search by a list of IDs.

Since lncRNASNP database contains data from human and mouse, we divided it into two sub-databases, namely lncRNASNP-human and lncRNASNP-mouse. Each of them displays data mainly through three sections, which are SNP section, lncRNA section and miRNA section.

The SNP section stored all the SNPs in lncRNAs as well as their detailed information. Data in this section include (i) basic information of the SNP; (ii) SNP impacts on lncRNA secondary structure; (iii) SNP impacts on miRNA–lncRNA interaction. It also contains information of SNPs in GWAS LD regions and related GWAS information (Figure 1B) in the lncRNASNP-human database. We set four options to optimize the SNP selection because of the huge SNP list. The first is the ‘position’ option allowing users to query SNPs in a user-defined chromosome region. The second is the ‘GMAF’ option helping users to filter results by the global minor allele frequency of a SNP, which is an important consideration of the sample size in association studies. Users can also limit the queried SNPs in GWAS regions or/and impacting miRNA–lncRNA interaction. By combining these options, users can quickly access to the interested polymorphisms.

The lncRNA section provides the information of lncRNAs. By clicking on a specific lncRNA, users can access its basic information, the SNPs on it, miRNAs binding to it and potential miRNA target sites gain/loss by SNPs (Figure 1C and E). In the ‘Basic information’ part, we provide the conservation information of lncRNA gene by integrating the results of Necsulea *et al*. (42) and lncRNAtor database (27). In the ‘miRNAs binding to lncRNA’ part, the potential binding miRNAs were shown by small bars on the binding location with different colors representing different target types (grouped by conservation, experimental evidence and miRNA expression level) (Figure 1E).

The miRNA section enables users to select interested miRNAs by expression to obtain their targeting lncRNAs and possible gain/loss target of lncRNAs by SNPs. In this section, the miRNAs have been classified into four categories according to their expressions. They were high expressed miRNA group (RPM ≥ 1000), middle expressed miRNA group (10 ≤ = RPM < 1000), low expressed miRNA group (1 ≤ RPM < 10) and unexpressed miRNA group (RPM < 1). For each human miRNA, we also provide the expression levels in 28 cancer types in the ‘basic information of miRNA’ part (Figure 1D).

## SUMMARY AND FUTURE DIRECTIONS

With the rapid advances in high-throughput technologies, the lncRNA identification increases greatly in the past few years. It is necessary to analyze the lncRNA related SNPs and construct a database to help elucidating the biological functions of lncRNAs. In this study, we systematically integrated lncRNA, miRNA and SNP information as well as GWAS results and miRNA expression profiles to seek potential functional SNPs. The lncRNASNP database can be used for either small or large-scale SNP selection from lncRNAs and is particularly useful for association and functional studies. In lncRNASNP database, we found 142 of GWAS tagSNPs in lncRNAs and lots of lncRNA SNPs in GWAS LD regions. These results will help to find the real causative SNPs and genes for GWAS studies. Furthermore, we have predicted the miRNA target sites on lncRNAs and integrated experimentally validated miRNA–lncRNA pairs in the database. In combination with other miRNA/gene databases, users can further explore the relationship between miRNA, lncRNA and protein-coding genes.

We can envision that in the next few years the number of lncRNAs will accumulate rapidly not only in human and mouse, but also in other species. In the future, lncRNASNP will update the database frequently and extend to other species. For other species without GWAS data, the eQTL data will be utilized for SNP selection. As the data accumulation of lncRNA expression and miRNA–lncRNA binding, we will update them into the database to reduce false positive predictions. We aim to develop and maintain the lncRNASNP database as a useful resource for the research community.
